# Clinical Outcomes of Extended-Release Guanfacine in Adults With Attention-Deficit/Hyperactivity Disorder: A Retrospective Chart Review

**DOI:** 10.7759/cureus.110044

**Published:** 2026-06-01

**Authors:** Hirohisa Suzuki, Wakaho Hayashi, Dan Nakamura, Shizuka Seki, Youichi Hanawa, Misato Yamauchi, Yoshifumi Nakamura, Shutaro Sugita, Kenji Sanada, Akira Iwanami

**Affiliations:** 1 Department of Psychiatry, Showa Medical University School of Medicine, Tokyo, JPN; 2 The Social, Genetic, and Developmental Psychiatry Centre, Institute of Psychiatry, Psychology, and Neuroscience, King's College London, London, GBR

**Keywords:** adult adhd, guanfacine, neurodevelopmental disorder, pharmacotherapy, real-world evidence

## Abstract

Introduction

Adult attention-deficit/hyperactivity disorder (ADHD) is a clinically important neurodevelopmental condition with a substantial global prevalence. Extended-release guanfacine (GXR) is a non-stimulant selective α2A-adrenergic receptor agonist approved for adult ADHD in Japan, but real-world adult data remain limited.

Materials and methods

We conducted a retrospective medical chart review of consecutive eligible adults with ADHD treated with GXR at an ADHD specialty clinic in Japan. ADHD was diagnosed according to the Diagnostic and Statistical Manual of Mental Disorders, Fifth Edition (DSM-5) criteria by experienced psychiatrists. Clinical Global Impression-Severity (CGI-S), GXR dose, treatment continuation at week 10, Clinical Global Impression-Improvement (CGI-I), and treatment-emergent adverse events (TEAEs) were extracted from medical records. Continuous variables were summarized as mean±standard deviation (SD) and categorical variables as number and percentage. Exact binomial 95% confidence intervals (CIs) were calculated for proportions. Exploratory baseline comparisons by treatment continuation status were performed using the Shapiro-Wilk test, Welch t-test, or Mann-Whitney U test for continuous variables and the Fisher exact test for categorical variables. Statistical analyses were performed using EZR, a graphical user interface for R.

Results

Among 137 participants, 111 (81%; 95% CI: 73.4-87.2%) continued GXR treatment for 10 weeks. Among the 111 participants with week-10 CGI-I data, 58 (52.3%; 95% CI: 42.6-61.8%) were rated as very much improved or much improved. TEAEs were recorded in 76 participants (55.5%; 95% CI: 46.7-64%), and no serious TEAEs were observed.

Conclusions

These findings suggest that GXR may be a clinically useful and generally well-tolerated treatment option for adults with ADHD in routine practice in Japan. The results support cautious real-world titration and highlight the need for prospective studies using standardized adult ADHD outcome measures.

## Introduction

Attention-deficit/hyperactivity disorder (ADHD) is a neurodevelopmental disorder characterized by developmentally inappropriate and impairing symptoms of inattention, hyperactivity, and impulsivity. A recent global systematic review and meta-analysis estimated the prevalence of persistent adult ADHD at 2.58% and symptomatic adult ADHD at 6.76%, indicating a substantial public health burden [1]. Adult ADHD is associated with impaired occupational and social functioning, psychiatric comorbidity, and reduced quality of life [2-6].

Psychostimulants such as methylphenidate and amphetamines have traditionally been central pharmacological treatments for ADHD. Extended-release guanfacine (GXR) is a non-stimulant selective α2A-adrenergic receptor agonist. In this context, "non-stimulant" indicates that the medication is not a psychostimulant such as methylphenidate or amphetamine, while "selective α2A-adrenergic receptor agonist" indicates the preferential stimulation of α2A-adrenoceptors, particularly postsynaptic receptors in prefrontal cortical networks implicated in attention, working memory, and behavioral regulation [7,8]. Guanfacine is thought to strengthen prefrontal cortical network connectivity by inhibiting cAMP-PKA signaling and reducing the opening of nearby potassium channels, thereby enhancing neuronal firing and top-down control [7].

In Japan, GXR is approved for adult ADHD, whereas approval for adult ADHD is not global. Japanese adult phase 3 trials have evaluated dose-optimized GXR in adults with ADHD, but real-world evidence remains limited [9-11]. The present retrospective chart review evaluated treatment continuation, clinical global improvement, dose distribution, and treatment-emergent adverse events (TEAEs) among adults with ADHD treated with GXR in routine clinical practice in Japan.

## Materials and methods

The study protocol was approved by the Showa Medical University Research Ethics Review Board Committee (approval number: 22-014-B), and participation was handled using an ethics committee-approved opt-out procedure.

This retrospective medical chart review was conducted in adults with ADHD who attended an ADHD specialty clinic at Showa Medical University Karasuyama Hospital in central Tokyo. The study period was from June 2019 to May 2022. Consecutive sampling was conducted: all adults who initiated GXR during the study period and met the inclusion and exclusion criteria were included in the analysis.

Eligible participants were adults aged 18 years or older who were diagnosed with ADHD according to the Diagnostic and Statistical Manual of Mental Disorders, Fifth Edition (DSM-5) [12], and who initiated GXR in routine clinical practice. ADHD diagnosis was made by experienced psychiatrists based on developmental history, current symptoms of inattention and/or hyperactivity-impulsivity, functional impairment, symptom onset history, and exclusion of alternative explanations for the symptoms. When available, developmental information from persons who had known the participant since childhood and school records were also considered. Exclusion criteria were an estimated premorbid IQ below 85, as assessed by the Japanese Adult Reading Test (JART) [13], age under 18 years, and the presence of other mental disorders or medical conditions judged to substantially confound the evaluation of GXR treatment outcomes.

No formal a priori sample size calculation was performed because this was a retrospective descriptive chart review. The sample size was determined by the number of consecutive eligible adults with ADHD who initiated GXR during the study period and had sufficient medical record data for the planned analyses.

Data were retrieved from hospital electronic medical records and extracted into Microsoft Excel (Microsoft Corporation, Redmond, Washington, United States) for data-entry checking and dataset preparation, with identifying information omitted. Participants were assigned random study identification numbers. Collected data included age, sex, educational history, estimated premorbid IQ, employment status, prior psychiatric consultation history, prior ADHD medication history, concomitant ADHD medications at GXR initiation, clinically recorded comorbid psychiatric diagnoses, baseline concomitant psychotropic medications, baseline Clinical Global Impression-Severity (CGI-S) score, GXR dose at initiation and week 10, treatment continuation status at week 10, discontinuation reason, week-10 Clinical Global Impression-Improvement (CGI-I) score, and TEAEs. Comorbid psychiatric diagnoses were extracted from clinically recorded diagnostic information in routine medical charts. Psychiatric symptoms or medication indications that were not recorded as formal comorbid diagnoses were not counted as comorbid psychiatric diagnoses. In this study, methylphenidate refers to extended-release methylphenidate prescribed for ADHD.

GXR dosing was determined by the treating psychiatrist as part of routine clinical care and was not specified by a study protocol. Although the labeled adult starting dose in Japan is 2 mg/day, some patients were started at 1 mg/day in routine practice based on the treating physician's judgment, considering factors such as body size, comorbidities, concomitant medications, baseline blood pressure or pulse rate, and concerns about sedation, hypotension, dizziness, or other tolerability issues.

Participants could be treatment-naïve or could have previously received other ADHD pharmacotherapies. GXR could be initiated either without concomitant ADHD medication or with concomitant ADHD medication in routine clinical practice. Medication changes before GXR initiation, including switching from or continuing other ADHD medications, were determined by the treating psychiatrist in routine clinical care. No protocol-defined washout period was required because this was a retrospective chart review, and concomitant ADHD medication use at GXR initiation was recorded.

Treatment continuation status, GXR dose, CGI-I, and TEAEs were evaluated at week 10. The main descriptive outcome was treatment continuation at week 10; additional descriptive outcomes included week-10 CGI-I, week-10 GXR dose distribution, discontinuation reasons, and TEAEs. The 10-week observation point was selected for this analysis because week-10 follow-up data were clinically available for evaluating early treatment continuation, dose titration, CGI-I, and TEAEs in routine practice and because this time point facilitated comparison with prior Japanese adult GXR trial data using a similar 10-week evaluation period.

The CGI-S and CGI-I were the clinician-rated scales used in this study. CGI-S is a single-item, 7-point global severity scale on which higher scores indicate greater illness severity, ranging from 1 (normal/not ill) to 7 (among the most extremely ill patients). CGI-I is a single-item, 7-point global improvement scale that assesses change relative to baseline, ranging from 1 (very much improved) to 7 (very much worse). These scales are widely used clinician global impression measures in psychiatric research and practice [14,15].

Statistical analysis

Continuous variables were summarized as mean±standard deviation (SD). Categorical variables were summarized as number and percentage. Exact binomial 95% confidence intervals (CIs) were calculated for proportions. For exploratory comparisons between participants who continued GXR treatment for 10 weeks and those who discontinued treatment before week 10, normality of continuous variables was assessed using the Shapiro-Wilk test. The Welch t-test or the Mann-Whitney U test was used for continuous variables, and the Fisher exact test was used for categorical variables, as appropriate. A two-sided p-value of <0.05 was considered statistically significant. No adjustment for multiple comparisons was performed. Statistical analyses were performed using EZR, a graphical user interface for R [16].

## Results

The study included 137 participants with a mean age of 35.8 years (SD 12.1), including 71 men (51.8%) and 66 women (48.2%). Demographic and clinical characteristics are summarized in Table 1.

**Table 1:** Demographic and clinical characteristics of the participants Data are presented as N (%) unless otherwise indicated. Prior ADHD medication, concomitant ADHD medication, concomitant non-ADHD psychotropic medication, and comorbid psychiatric diagnosis categories may overlap, except for no prior ADHD medication, no concomitant ADHD medication, and no concomitant non-ADHD psychotropic medication. Psychotropic medication use was recorded separately from formally documented comorbid psychiatric diagnoses and should not be interpreted as evidence of a formal comorbid diagnosis. Methylphenidate refers to extended-release methylphenidate prescribed for ADHD. Clinically recorded comorbid psychiatric diagnoses refer to formal diagnoses documented in routine medical charts; psychiatric symptoms or medication indications not recorded as formal comorbid diagnoses were not counted. ADHD: attention-deficit/hyperactivity disorder; GXR: extended-release guanfacine

Variable	Values (mean±SD/N (%))
Total participants	137
Age, years	35.8±12.1
Estimated premorbid IQ	103.0±12.5
Sex	
Men	71 (51.8%)
Women	66 (48.2%)
Employment status	
Full-time worker	55 (40.1%)
Part-time worker	19 (13.9%)
Student or housewife	34 (24.8%)
Unemployed	29 (21.2%)
Previous psychiatric consultation history	
Outpatient treatment only	96 (70.1%)
History of hospitalization	7 (5.1%)
No previous psychiatric consultation	34 (24.8%)
Prior ADHD medication history	
Prior atomoxetine	63 (46%)
Prior methylphenidate	44 (32.1%)
Prior both atomoxetine and methylphenidate	13 (9.5%)
No prior ADHD medication	43 (31.4%)
Concomitant ADHD medications at GXR initiation	
Any concomitant ADHD medication	41 (29.9%)
Concomitant atomoxetine	30 (21.9%)
Concomitant methylphenidate	22 (16.1%)
Concomitant atomoxetine and methylphenidate	11 (8%)
No concomitant ADHD medication	96 (70.1%)
Baseline concomitant non-ADHD psychotropic medications	
Any concomitant non-ADHD psychotropic medication	76 (55.5%)
Concomitant antidepressants	39 (28.5%)
Concomitant mood stabilizers	22 (16.1%)
Concomitant antipsychotics	15 (10.9%)
Concomitant anxiolytics	35 (25.5%)
Concomitant hypnotics	28 (20.4%)
No concomitant non-ADHD psychotropic medication	61 (44.5%)
Clinically recorded comorbid psychiatric diagnoses	
Any clinically recorded comorbid psychiatric diagnosis	19 (13.9%)
Depression	12 (8.8%)
Anxiety disorder	4 (2.9%)
Eating disorder	2 (1.5%)
Personality disorder	2 (1.5%)
Substance use disorder	1 (0.7%)
Educational history	
Years of education	14.5±2.3
History of alcohol misuse	
Yes	3 (2.2%)
No	134 (97.8%)
History of illicit drug use	
Yes	4 (2.9%)
No	133 (97.1%)
Family history of psychiatric disorders	
Yes	65 (47.4%)
No	72 (52.6%)

Baseline characteristics stratified by week-10 treatment continuation status are shown in Table 2.

**Table 2:** Baseline characteristics by week-10 treatment continuation status Data are presented as mean±SD or N (%). Continuous variables were compared using the Welch t-test or Mann-Whitney U test and categorical variables using the Fisher exact test. P-values were not adjusted for multiple comparisons and should be interpreted as exploratory. Medication history and concomitant medication use are summarized separately in Table 1 and Figure 1. CGI-S: Clinical Global Impression-Severity

Characteristic	Overall (N=137)	Continued (N=111)	Discontinued (N=26)	Test	P-value
Age, years	35.8±12.1	35.2±11.2	38.2±14.0	Welch t-test	0.316
Education, years	14.5±2.3	14.8±2.1	13.7±2.8	Welch t-test	0.069
Estimated premorbid IQ	103.0±12.5	103.7±11.6	100.0±15.2	Welch t-test	0.253
Baseline CGI-S	3.8±0.8	3.7±0.7	4.0±0.9	Mann-Whitney U test	0.064
Female sex	66 (48.2)	51 (45.9)	15 (57.7)	Fisher exact test	0.383
Clinically recorded psychiatric comorbidity	19 (13.9)	17 (15.3)	2 (7.7)	Fisher exact test	0.528

**Figure 1:**
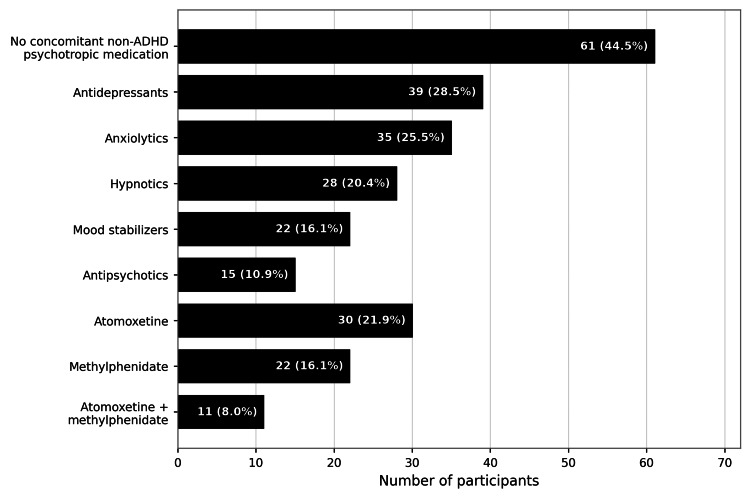
Concomitant medications at the start of GXR treatment Bars show the number and percentage of participants receiving each medication category at the initiation of GXR treatment. Medication categories were not mutually exclusive. ADHD medications prescribed concomitantly at GXR initiation included atomoxetine, methylphenidate, and combined atomoxetine plus methylphenidate. The no concomitant non-ADHD psychotropic medication category refers to the absence of concomitant antidepressants, anxiolytics, hypnotics, mood stabilizers, or antipsychotics and did not exclude concomitant ADHD medication use. Methylphenidate refers to extended-release methylphenidate prescribed for ADHD. ADHD: attention-deficit/hyperactivity disorder; GXR: extended-release guanfacine

In this exploratory comparison, no statistically significant differences were observed for the demographic and clinical baseline variables shown in Table 2. These comparisons were not adjusted for multiple testing and were based on imbalanced group sizes; therefore, they should be interpreted as descriptive and hypothesis-generating rather than confirmatory. Baseline medication history is summarized in Table 1, and concomitant medications at GXR initiation, including concomitant ADHD medications, are presented in Figure 1.

**Figure 2:**
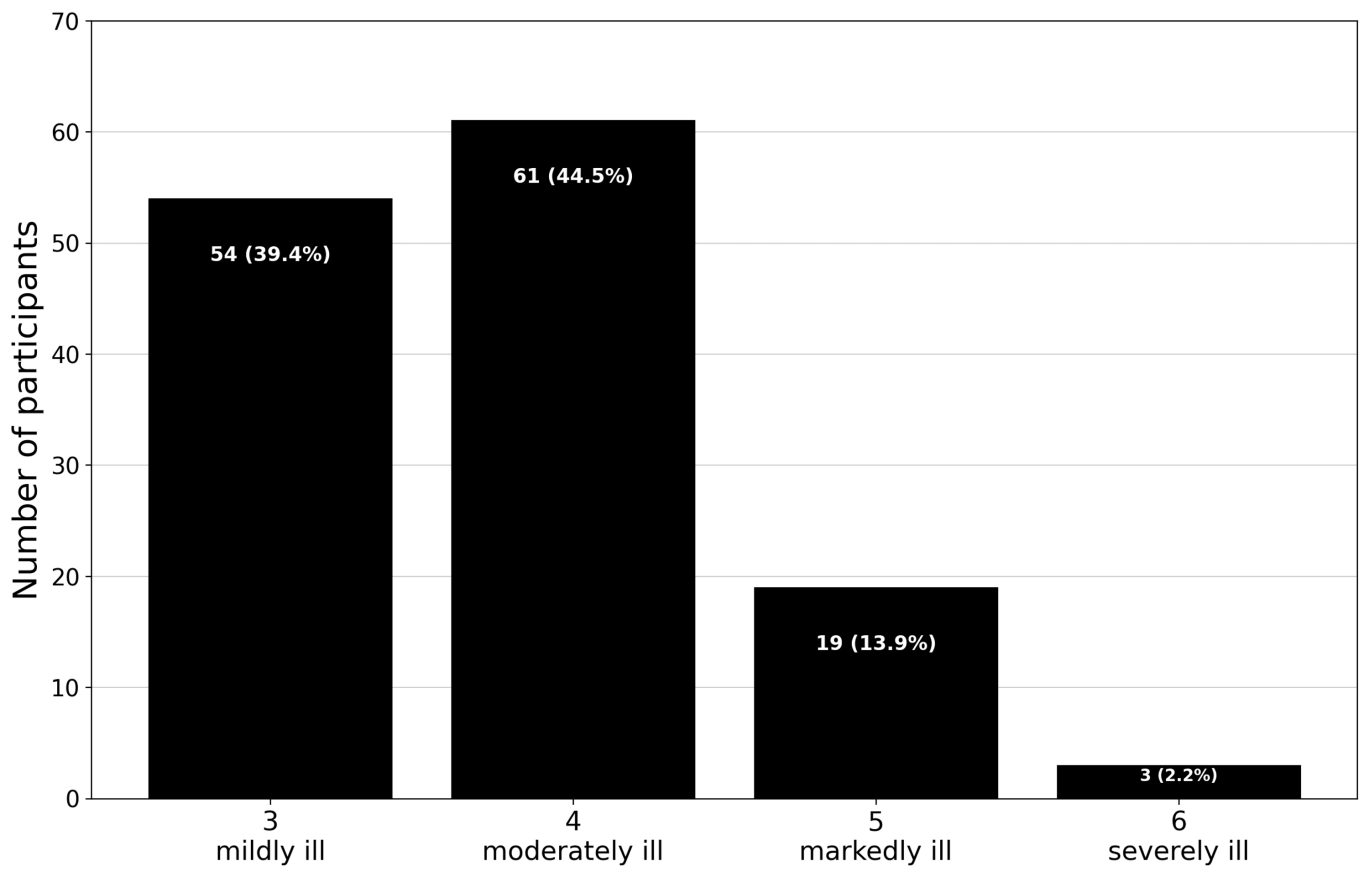
CGI-S score at baseline CGI-S is a 7-point clinician-rated global severity scale. Data are presented as N (%) among all participants (N=137). CGI-S: Clinical Global Impression-Severity

Antidepressants were used by 39 participants (28.5%) and anxiolytics by 35 (25.5%), making these the most frequently used non-ADHD psychotropic medications. Among concomitant ADHD medications at GXR initiation, atomoxetine was used by 30 participants (21.9%), methylphenidate by 22 (16.1%), and combined atomoxetine plus methylphenidate by 11 (8%). Medication categories were not mutually exclusive. Baseline CGI-S score distribution is shown in Figure 2.

**Figure 3:**
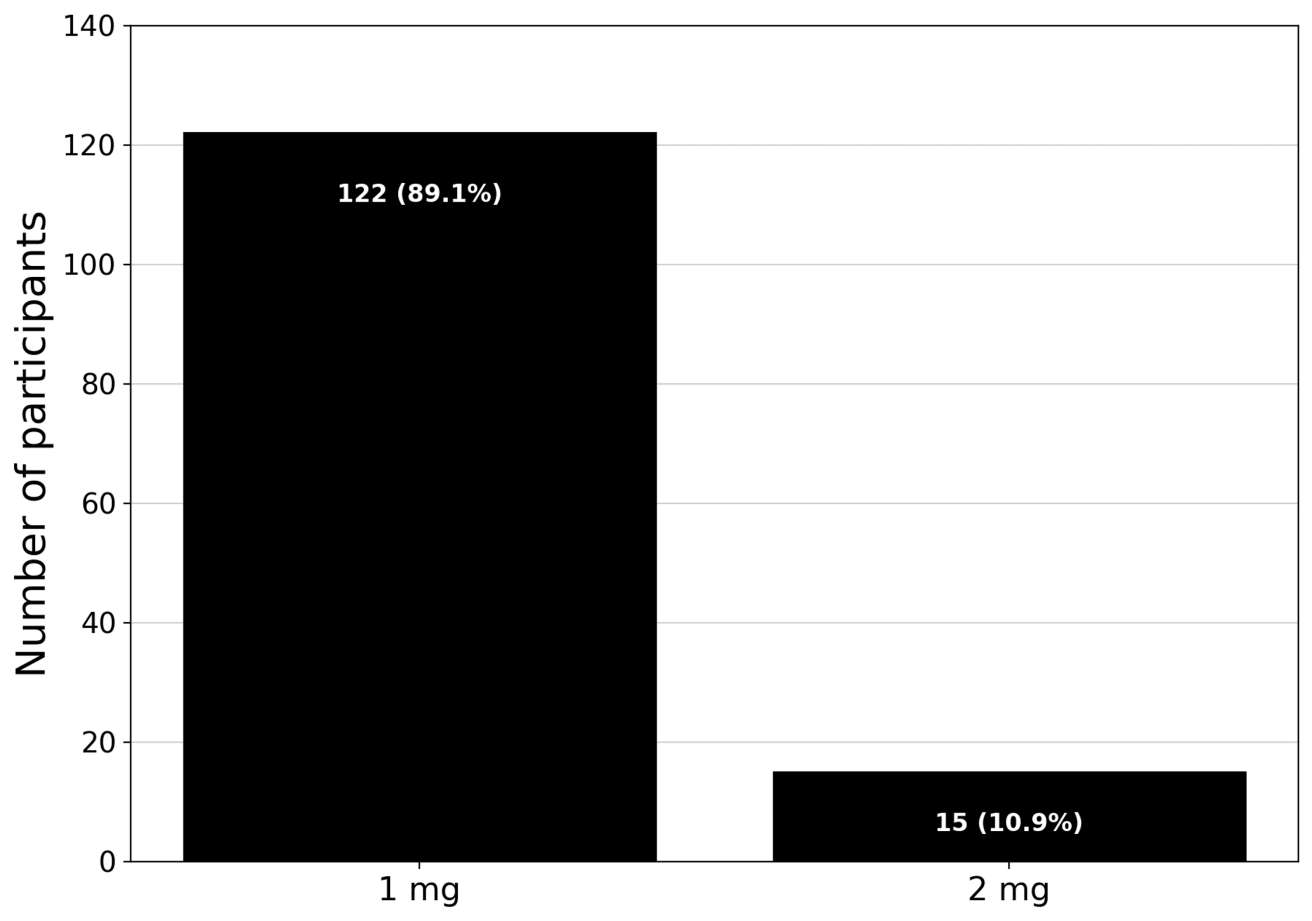
Baseline dose of GXR (mg) Data are presented as N (%) among all participants (N=137): 1 mg, 122 (89.1%); 2 mg, 15 (10.9%). GXR: extended-release guanfacine

Fifty-four participants (39.4%) had a score of 3, 61 (44.5%) had a score of 4, 19 (13.9%) had a score of 5, and three (2.2%) had a score of 6. Baseline GXR dose distribution is shown in Figure 3.

One hundred and twenty-two participants (89.1%) received 1 mg, and 15 (10.9%) received 2 mg.

Among the 137 participants, 111 (81%; 95% CI: 73.4-87.2%) continued GXR treatment for 10 weeks. The reasons for discontinuation included side effects in 12 cases (8.8%; 95% CI: 4.6-14.8%), insufficient efficacy in six cases (4.4%; 95% CI: 1.6-9.3%), and other reasons in eight cases (5.8%; 95% CI: 2.6-11.2%).

Continuous findings presented in the Results are summarized in Table 3.

**Table 3:** Continuous findings presented in the Results Continuous values are presented as mean±SD. Week-10 values are summarized among participants with available week-10 data. CGI-I: Clinical Global Impression-Improvement; CGI-S: Clinical Global Impression-Severity; GXR: extended-release guanfacine

Variable	Analysis set	N	Mean±SD	Median	IQR	Range
Age, years	All participants	137	35.8±12.1	35	27-42	18-70
Education, years	All participants	137	14.5±2.3	15	13-16	9-22
Estimated premorbid IQ	All participants	137	103.0±12.5	102.8	94.2-112.4	88.0-132.1
Baseline CGI-S	All participants	137	3.8±0.8	4	3.0-4.0	3.0-6.0
Baseline GXR dose, mg/day	All participants	137	1.1±0.3	1	1.0-1.0	1.0-2.0
Week-10 GXR dose, mg/day	Participants with week-10 data	111	2.7±1.2	3	2.0-3.0	1.0-6.0
Week-10 CGI-I	Participants with week-10 data	111	2.5±0.8	2	2.0-3.0	1.0-4.0

Key categorical outcomes with exact binomial 95% CIs are summarized in Table 4.

**Table 4:** Highlighted categorical outcomes with exact binomial 95% CI In the all-treated/nonresponder sensitivity analysis, participants who discontinued GXR before week 10 were treated as nonresponders. CGI-I: Clinical Global Impression-Improvement; GXR: extended-release guanfacine; TEAE: treatment-emergent adverse event

Outcome	n	Denominator	%	95% CI
Continued GXR treatment for 10 weeks	111	137	81	73.4-87.2
Discontinued before week 10	26	137	19	12.8-26.6
Discontinued because of side effects	12	137	8.8	4.6-14.8
Discontinued because of insufficient efficacy	6	137	4.4	1.6-9.3
Discontinued for other reasons	8	137	5.8	2.6-11.2
CGI-I score 1 or 2 at week 10	58	111	52.3	42.6-61.8
CGI-I score 1 or 2 at week 10, all-treated/nonresponder sensitivity analysis	58	137	42.3	33.9-51.1
Any TEAE	76	137	55.5	46.7-64.0
Serious TEAE	0	137	0	0.0-2.7
No TEAEs recorded	61	137	44.5	36.0-53.3

**Figure 4:**
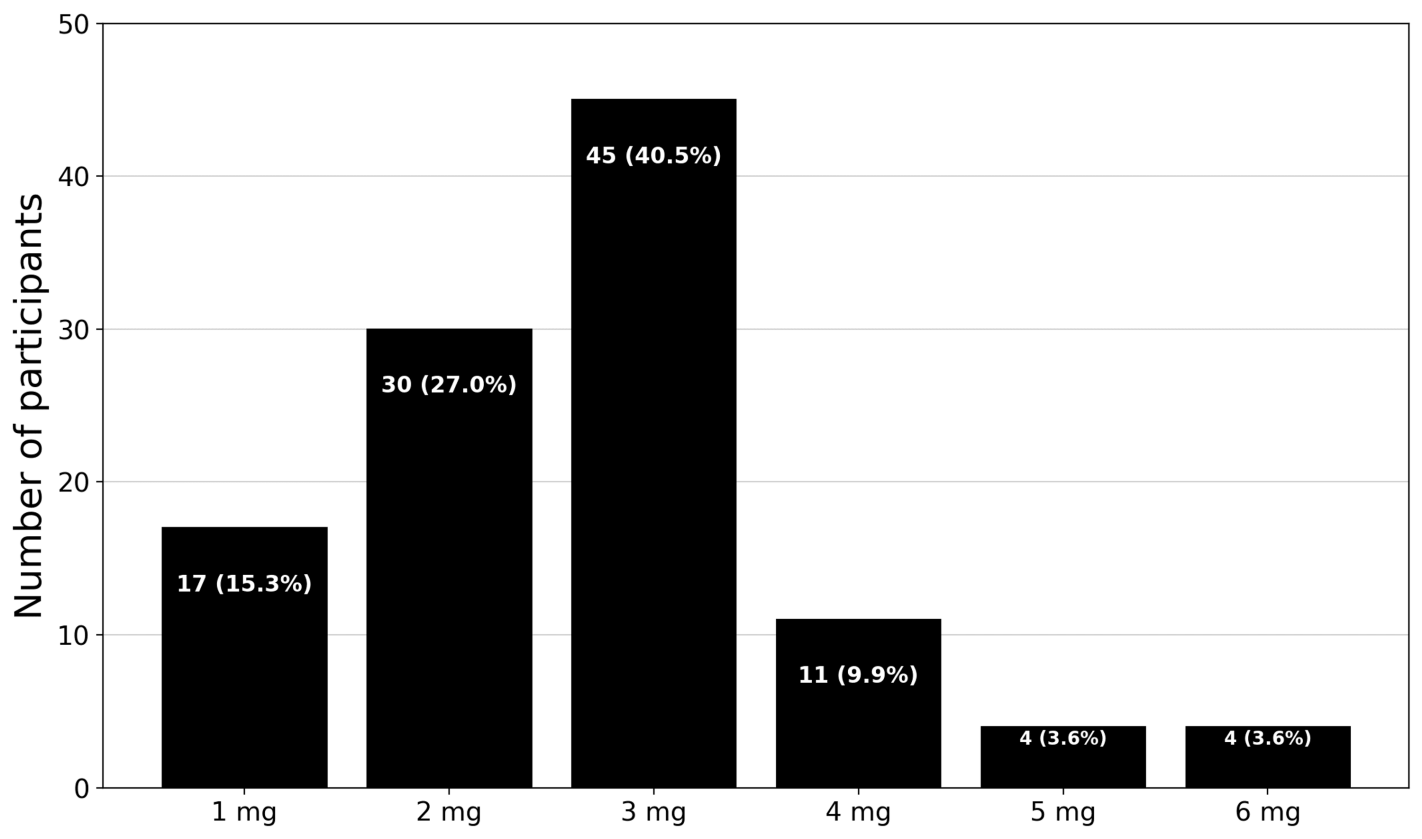
Dose of GXR (mg) at week 10 Data are presented as N (%) among participants with available week-10 dose data (N=111). GXR: extended-release guanfacine

Week-10 GXR dose distribution is shown in Figure 4 and Table 5, and the corresponding mean dose is summarized in Table 3.

**Table 5:** Week-10 dose distribution of GXR with exact binomial 95% CI Data are presented among participants with available week-10 dose data (N=111). GXR: extended-release guanfacine

GXR dose at week 10	n	Denominator	%	95% CI
1 mg	17	111	15.3	9.2-23.4
2 mg	30	111	27	19.0-36.3
3 mg	45	111	40.5	31.3-50.3
4 mg	11	111	9.9	5.1-17.0
5 mg	4	111	3.6	1.0-9.0
6 mg	4	111	3.6	1.0-9.0

**Figure 5:**
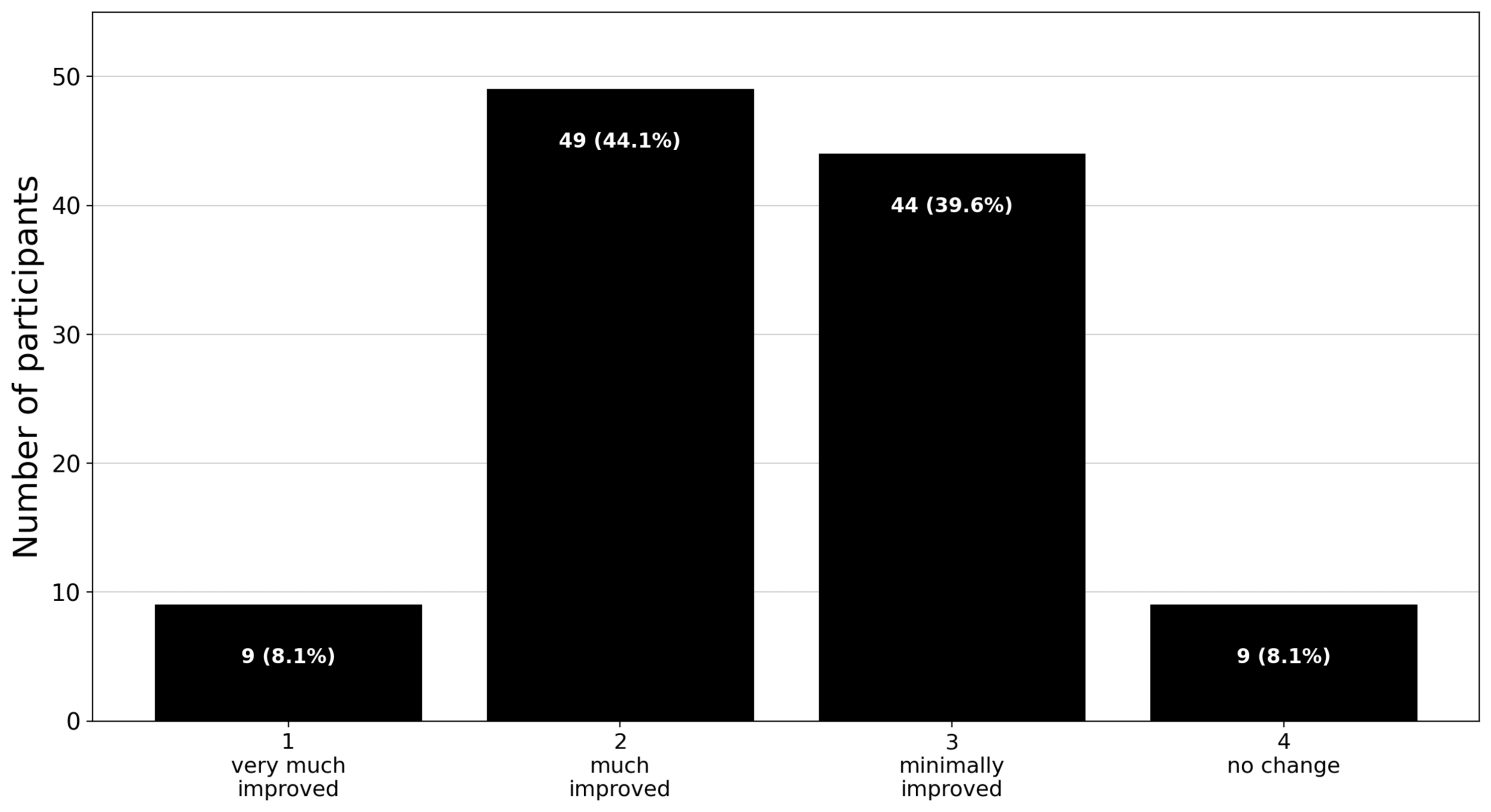
CGI-I score at week 10 CGI-I is a 7-point clinician-rated global improvement scale. Data are presented as N (%) among participants with available week-10 CGI-I data (N=111). Scores of 5-7 did not occur. CGI-I: Clinical Global Impression-Improvement

The mean dose at week 10 was 2.7 mg (SD 1.2), and 3 mg was the most frequently prescribed week-10 dose. Week-10 CGI-I scores are shown in Figure 5 and Table 6.

**Table 6:** CGI-I distribution at week 10 with exact binomial 95% CI CGI-I: Clinical Global Impression-Improvement

CGI-I score	Interpretation	n	Denominator	%	95% CI
1	Very much improved	9	111	8.1	3.8-14.8
2	Much improved	49	111	44.1	34.7-53.9
3	Minimally improved	44	111	39.6	30.5-49.4
4	No change	9	111	8.1	3.8-14.8
5	Minimally worse	0	111	0	0.0-3.3
6	Much worse	0	111	0	0.0-3.3
7	Very much worse	0	111	0	0.0-3.3

Nine participants (8.1%) were rated as 1, 49 (44.1%) as 2, 44 (39.6%) as 3, and nine (8.1%) as 4. Scores of 5-7 were not observed. The mean CGI-I score was 2.5 (SD 0.8), and 58 of the 111 participants (52.3%; 95% CI: 42.6-61.8%) were rated as very much improved or much improved. In a sensitivity analysis in which all 26 participants who discontinued GXR before week 10 were treated as nonresponders, 58 of all 137 participants (42.3%; 95% CI: 33.9-51.1%) were classified as very much improved or much improved.

**Figure 6:**
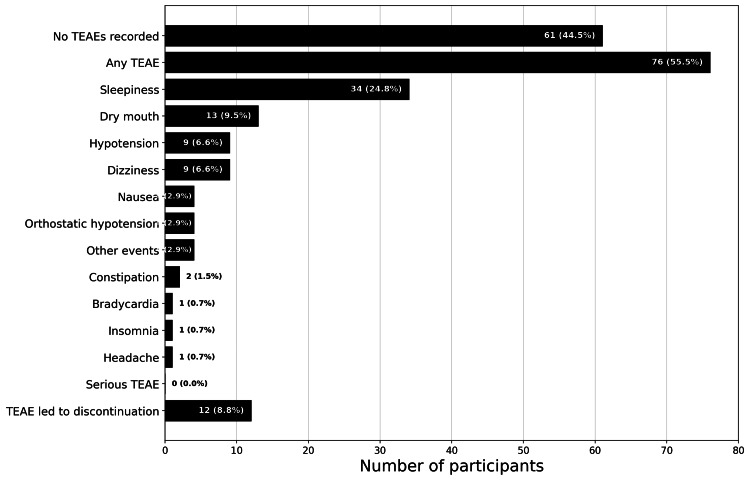
TEAEs during the 10-week observation period Data are presented as N (%) among all participants (N=137). TEAE categories are not mutually exclusive. "No TEAEs recorded" indicates participants with no TEAEs recorded during the observation period. TEAE: treatment-emergent adverse event

TEAEs are shown in Figure 6 and Table 7.

**Table 7:** TEAEs with 95% CI TEAE categories are not mutually exclusive. "No TEAEs recorded" indicates participants with no TEAEs recorded during the observation period. TEAE: treatment-emergent adverse event

TEAE category	n	Denominator	%	95% CI
No TEAEs recorded	61	137	44.5	36.0-53.3
Any TEAE	76	137	55.5	46.7-64.0
Sleepiness	34	137	24.8	17.8-32.9
Dry mouth	13	137	9.5	5.1-15.7
Hypotension	9	137	6.6	3.0-12.1
Dizziness	9	137	6.6	3.0-12.1
Nausea	4	137	2.9	0.8-7.3
Orthostatic hypotension	4	137	2.9	0.8-7.3
Other events	4	137	2.9	0.8-7.3
Constipation	2	137	1.5	0.2-5.2
Bradycardia	1	137	0.7	0.0-4.0
Insomnia	1	137	0.7	0.0-4.0
Headache	1	137	0.7	0.0-4.0
Serious TEAE	0	137	0	0.0-2.7
TEAE led to discontinuation	12	137	8.8	4.6-14.8

**Figure 7:**
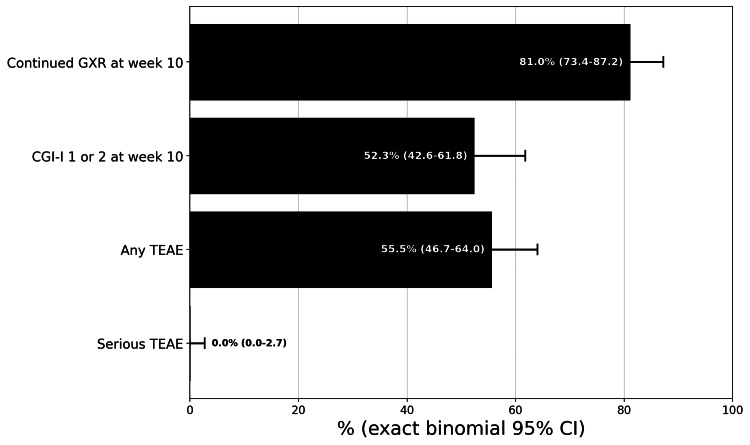
Highlighted findings with 95% CI Percentages and exact binomial 95% CI are shown for treatment continuation, CGI-I response, any TEAE, and serious TEAE. TEAE: treatment-emergent adverse event; GXR: extended-release guanfacine; CGI-I: Clinical Global Impression-Improvement

Overall, 82 TEAE category entries were recorded among 76 participants (55.5%; 95% CI: 46.7-64%). The most common TEAEs were sleepiness, dry mouth, hypotension, and dizziness. No serious TEAEs were reported. The highlighted findings are also presented graphically with exact binomial 95% CIs in Figure 7.

## Discussion

In this retrospective chart review of adults with ADHD treated with GXR in routine clinical practice in Japan, 81% of participants continued treatment for 10 weeks, 52.3% of those with week-10 CGI-I data were rated as very much improved or much improved, and no serious TEAEs were observed. These findings are broadly consistent with Japanese adult trial data and suggest that GXR may be clinically useful in adult ADHD under real-world conditions [9,10].

The pharmacological rationale for GXR differs from that of psychostimulants. Guanfacine is a selective α2A-adrenergic receptor agonist that is thought to act on prefrontal cortical circuits involved in attention, working memory, and behavioral inhibition. By stimulating postsynaptic α2A-adrenoceptors, guanfacine may reduce cAMP-PKA signaling and strengthen prefrontal network connectivity, which provides a plausible mechanism for improvement in ADHD symptoms [7,8].

The Japanese recommended adult dosage for GXR is a usual starting dose of 2 mg/day, titration by 1 mg at intervals of at least one week, a maintenance dose of 4-6 mg/day, and a maximum daily dose of 6 mg administered orally once daily [11]. In the present real-world cohort, most participants started at 1 mg, and the mean week-10 dose was lower than the recommended maintenance range. This likely reflects cautious titration in routine practice, possibly due to concerns about tolerability, comorbidities, concomitant medications, baseline blood pressure or pulse rate, or sedation and dizziness. Therefore, discontinuation due to insufficient efficacy should be interpreted cautiously, because some patients may not have reached the recommended maintenance dose during the 10-week observation period. The present findings should not be interpreted as evidence that a mean week-10 dose of 2.7 mg/day represents an optimal maintenance dose for adults with ADHD.

Clinically relevant precautions for GXR include sedation, hypotension, bradycardia, syncope, renal or hepatic impairment, abrupt discontinuation, and interactions with strong CYP3A4 inhibitors or inducers [8,11]. The reported elimination half-life of approximately 16-17 hours supports once-daily administration [8]. Previous adult GXR studies reported TEAEs such as somnolence, thirst or dry mouth, decreased blood pressure, postural dizziness, constipation, and related cardiovascular effects, with most TEAEs being mild to moderate [9,10]. Adult evidence for guanfacine outside the Japanese phase 3 program has remained limited, with prior guideline, review, and small adult study literature providing only limited support for adult use [17-21]. Pediatric and adolescent studies have also reported somnolence/sedation, fatigue, headache, gastrointestinal symptoms, decreased blood pressure, and bradycardia as clinically relevant adverse effects [22-25]. Although guanfacine has been discussed in relation to other conditions involving prefrontal cortical dysfunction, the present study evaluated its use for adult ADHD in Japan and does not provide evidence for off-label indications [7,8].

The exploratory comparison by treatment continuation status did not identify statistically significant differences in the demographic and clinical baseline variables shown in Table 2, including age, education years, estimated premorbid IQ, baseline CGI-S, sex, and clinically recorded psychiatric comorbidity. However, because these comparisons were not adjusted for multiple testing and the discontinued group was small, these findings should be considered descriptive and hypothesis-generating rather than confirmatory. Baseline medication history and concomitant medication use were summarized separately in Table 1 and Figure 1, and medication-related confounding remains an important limitation of this retrospective study.

This study provides real-world data from a specialized adult ADHD clinic in Japan, including treatment continuation, dose distribution, clinician-rated global severity and improvement, TEAEs, and confidence intervals for key categorical outcomes. The findings suggest that GXR may be a useful non-stimulant option for adults with ADHD, including patients who require alternatives to or augmentation of other ADHD treatments. The observed dosing pattern also highlights that cautious initiation and gradual titration are common in routine practice. Future prospective multicenter studies using standardized adult ADHD symptom scales, patient-reported outcomes, adherence measures, and comparative designs are needed to clarify optimal dosing, clinical outcomes, tolerability, and predictors of continuation.

In addition, prior longitudinal and clinical studies have reported that adult ADHD symptoms may persist from childhood into adulthood and may be associated with depressive symptoms, impaired quality of life, psychosocial adversity, mortality, and suicidal behavior, reinforcing the clinical relevance of real-world adult ADHD treatment research [26-30].

This study should be interpreted in light of several limitations. First, because this was a retrospective analysis of medical records without a control group and with a relatively short observation period, causal interpretation is limited. The participants were recruited from a single ADHD specialty clinic, and the sample size was relatively small; therefore, generalizability to broader adult ADHD populations, including general psychiatric clinics and primary care settings, may be limited. The timing of routine follow-up visits around week 10 was not protocol-scheduled, and variation in visit timing may have affected week-10 dose, CGI-I, and TEAE assessment.

Second, week-10 CGI-I outcomes were evaluated only among participants who continued GXR treatment until week 10 and had available week-10 CGI-I data. Therefore, the CGI-I findings represent a completer analysis and may overestimate clinical improvement because participants who discontinued treatment before week 10, including those who discontinued because of insufficient efficacy or adverse events, were not included in the week-10 CGI-I analysis. Because last-observation-carried-forward data were not consistently available in the retrospective charts, an intention-to-treat analysis of CGI-I outcomes could not be performed.

Third, CGI-I was assessed in routine, open-label clinical practice and was based on clinician global judgment rather than blinded assessment or standardized adult ADHD-specific symptom scales. The absence of CGI-I worsening scores among participants with week-10 data should therefore be interpreted cautiously, as observer bias may have influenced CGI-I ratings.

Fourth, standardized adult ADHD-specific symptom rating scales, such as the Adult ADHD Self-Report Scale (ASRS) or Conners' Adult ADHD Rating Scales (CAARS), and structured diagnostic interviews, such as the Diagnostic Interview for ADHD in Adults (DIVA) or Conners' Adult ADHD Diagnostic Interview for DSM-IV (CAADID), were not uniformly available in the retrospective charts. Although diagnoses were made by experienced psychiatrists according to DSM-5 criteria using developmental and clinical history, the lack of standardized ADHD-specific diagnostic and outcome measures limits the objectivity, reproducibility, and interpretability of the findings.

Fifth, comorbid psychiatric diagnoses were extracted from clinically recorded diagnostic information in routine medical charts and may have been under-recorded. Reliance on formally documented comorbid diagnoses may therefore have led to the underestimation of the true prevalence of psychiatric comorbidity, and the number of recorded comorbid psychiatric diagnoses should not be interpreted as equivalent to the frequency of clinically relevant psychiatric symptoms or psychotropic medication use. Concomitant psychotropic medications may have been prescribed symptomatically for insomnia, anxiety, depressive symptoms, emotional dysregulation, irritability, or other symptoms encountered in routine ADHD care without a formally recorded comorbid psychiatric diagnosis. This discrepancy limits the interpretation of baseline psychiatric comorbidity and may reduce the generalizability of the findings.

In addition, because this was a retrospective chart review of routine clinical practice, medication switching and washout procedures before GXR initiation were not standardized by protocol. Residual effects of prior ADHD medications, effects of concomitant ADHD medications, or symptoms related to medication switching, therefore, could not be fully separated from the effects of GXR.

Sixth, baseline medication history and concomitant medication use were summarized descriptively, but medication-related baseline comparisons and medication-change analyses during the 10-week observation period were limited. The present study did not perform outcome analyses stratified by concomitant ADHD medication status. Therefore, we could not determine whether outcomes differed between GXR monotherapy and adjunctive GXR use with atomoxetine or methylphenidate. In addition, changes in concomitant medications during the 10-week observation period, including ADHD medications and non-ADHD psychotropic medications, dose adjustments, or newly added medications, were not systematically controlled or analyzed; therefore, such changes may have confounded CGI-I improvement and tolerability outcomes.

Seventh, because this was a study of real-world clinical practice, medication adherence, access to care, comorbidities, and concomitant medications may have affected the results. TEAEs may also have been under-recorded in routine medical charts.

Finally, estimated premorbid IQ was assessed using the JART rather than a full standardized intelligence test such as the Wechsler Adult Intelligence Scale (WAIS). Although the JART is a practical tool for estimating premorbid intellectual ability in routine clinical settings, it does not provide a comprehensive assessment of current cognitive functioning, working memory, processing speed, or cognitive profile. Therefore, intellectual and neuropsychological heterogeneity among adults with ADHD may not have been fully captured.

## Conclusions

In this retrospective medical chart review, most adults with ADHD who initiated GXR continued treatment for 10 weeks, more than half of those with week-10 CGI-I data were rated as very much improved or much improved, and no serious TEAEs were observed. These findings provide useful real-world evidence supporting the potential clinical utility and tolerability of GXR in adults with ADHD in Japan. Because the study was retrospective and uncontrolled, prospective multicenter studies using standardized adult ADHD outcome measures are needed to further evaluate optimal dosing, clinical outcomes, tolerability, and predictors of continuation.
